# Downregulation of Snail by DUSP1 Impairs Cell Migration and Invasion through the Inactivation of JNK and ERK and Is Useful as a Predictive Factor in the Prognosis of Prostate Cancer

**DOI:** 10.3390/cancers13051158

**Published:** 2021-03-08

**Authors:** Desirée Martínez-Martínez, María-Val Toledo Lobo, Pablo Baquero, Santiago Ropero, Javier C. Angulo, Antonio Chiloeches, Marina Lasa

**Affiliations:** 1 Departamento de Bioquímica-Instituto de Investigaciones Biomédicas “Alberto Sols”, Universidad Autónoma de Madrid-Consejo Superior de Investigaciones Científicas, E-28029 Madrid, Spain; desiree.martinez@edu.uah.es; 2 Departamento de Biomedicina y Biotecnología, Universidad de Alcalá, E-28805 Madrid, Spain; mval.toledo@uah.es; 3 IRYCIS, Instituto de Investigaciones Sanitarias Ramón y Cajal, E-28034 Madrid, Spain; 4 Departamento de Biología de Sistemas, Unidad de Bioquímica y Biología Molecular, Facultad de Medicina, Universidad de Alcalá, E-28805 Madrid, Spain; pablo.baquero@uah.es (P.B.); santiago.ropero@uah.es (S.R.); antonio.chiloeches@uah.es (A.C.); 5 Servicio de Urología, Hospital Universitario de Getafe, E-28905 Madrid, Spain; javier.angulo@salud.madrid.org

**Keywords:** DUSP1, MAPK, Snail, prostate cancer, migration and invasion, patient survival, biomarkers

## Abstract

**Simple Summary:**

The role of dual specificity phosphatase 1 (DUSP1) in metastasis-associated processes in prostate cancer and its impact on patient outcome remains to be elucidated. Our results reveal that this phosphatase reduces Snail expression and impairs cell migration and invasion in prostate cancer cells through a mechanism involving the inhibition of DUSP1 molecular targets, c-Jun N-terminal kinase (JNK) and extracellular-signal-regulated kinase (ERK). In clinical samples, we evidence an inverse correlation between DUSP1 expression and Snail levels, which are further associated with JNK and ERK activation. Importantly, patients with the pattern DUSP1_high_/activated JNK_low_/activated ERK_low_/Snail_low_ exhibit a longer time to progression and a better outcome than those with the opposite pattern. All these findings highlight new opportunities to improve current therapeutic strategies for the diagnosis and treatment of prostate cancer.

**Abstract:**

Dual specificity phosphatase 1 (DUSP1) is crucial in prostate cancer (PC), since its expression is downregulated in advanced carcinomas. Here, we investigated DUSP1 effects on the expression of mesenchymal marker Snail, cell migration and invasion, analyzing the underlying mechanisms mediated by mitogen-activated protein kinases (MAPKs) inhibition. To this purpose, we used different PC cells overexpressing or lacking DUSP1 or incubated with MAPKs inhibitors. Moreover, we addressed the correlation of DUSP1 expression with Snail and activated MAPKs levels in samples from patients diagnosed with benign hyperplasia or prostate carcinoma, studying its implication in tumor prognosis and survival. We found that DUSP1 downregulates Snail expression and impairs migration and invasion in PC cells. Similar results were obtained following the inhibition of c-Jun N-terminal kinase (JNK) and extracellular-signal-regulated kinase (ERK). In clinical samples, we evidenced an inverse correlation between DUSP1 expression and Snail levels, which are further associated with JNK and ERK activation. Consequently, the pattern DUSP1_high_/activated JNK_low_/activated ERK_low_/Snail_low_ is associated with an overall extended survival of PC patients. In summary, the ratio between DUSP1 and Snail expression, with additional JNK and ERK activity measurement, may serve as a potential biomarker to predict the clinical outcome of PC patients. Furthermore, DUSP1 induction or inhibition of JNK and ERK pathways could be useful to treat PC.

## 1. Introduction

Prostate cancer is one of the most frequently diagnosed cancers in men worldwide and is the second leading cause of cancer-related deaths among males [1]. The majority of the deaths associated with this type of tumors are related to metastasis, in which the so-called epithelial–mesenchymal transition (EMT) is one of the most important events involved [2]. EMT is a cell plasticity program that plays very important roles during embryonic development and can be reactivated in adult physiological situations to maintain epithelial homeostasis in order to guarantee tissue integrity and organ function [3,4]. Moreover, EMT also has important roles in pathological processes such as cancer metastasis. This process is defined by a loss of epithelial cell-specific characteristics, such as polarity and cohesiveness, and by an acquisition of a mesenchymal-like morphology with increased motility [5]. The abnormal activation of EMT in cancer disrupts the intercellular junctions, causing the dissociation of surrounding cells and the acquisition of migratory phenotype. Thus, EMT is often associated with the invasion and metastatic ability of tumor cells. In agreement with this, a large amount of evidence have shown that metastatic cells display a decreased expression of epithelial markers and an increased expression of mesenchymal markers both in vitro and in vivo [4]. One of the hallmarks of the EMT is the overexpression of Snail, which is a transcription factor that downregulates the expression of epithelial genes and upregulates the expression of mesenchymal genes, ultimately leading to increased migration and invasion [6]. Thus, Snail overexpression has been found in the invasive fronts of several human tumors derived from epithelial cells, including hepatocellular, breast, or thyroid carcinomas, among others [7,8,9,10,11]. Accordingly, Snail is widely associated with invasiveness, metastasis, tumor recurrence, and poor prognosis [7,8,9]. In particular, metastatic prostate cancer cells display typical features of EMT, and Snail plays an important role in the regulation of cell polarity, the expression of epithelial and mesenchymal markers, as well as migration and invasion [2,12]. Consistently, Snail expression increases with prostate cancer progression from benign to bone metastatic tumors [13,14,15]. From a molecular point of view, several studies in different tumor contexts have demonstrated that the expression and activity of Snail can be regulated by multiple molecular mechanisms, including transcriptional regulation and post-translational modifications. In this sense, one of the most important mechanisms that affects Snail stability involves its export from the nucleus and its subsequent degradation by the proteasome in the cytosol [16]. Furthermore, it has been demonstrated that mitogen-activated protein kinase (MAPK) activation results in an increase of Snail protein levels, which in turn regulate the expression of EMT-associated genes [16].

Dual specificity phosphatase 1 (DUSP1) acts as a tumor suppressor by negatively regulating MAPK activity in different tumors, including prostate cancer. Thus, we and others have previously demonstrated that the expression of this phosphatase decreases with prostate tumor progression. Whereas DUSP1 levels are high in benign prostatic hyperplasia (BPH) and hormone-sensitive prostatic adenocarcinoma (HS-PC), the expression of this phosphatase is almost absent in hormone-refractory prostatic adenocarcinoma (HR-PC) [17,18]. Consistently, DUSP1 overexpression in androgen-independent prostate cancer cells promotes apoptosis through inhibition of the p38 mitogen-activated protein kinase (p38MAPK)/nuclear factor-kappaB (NF-kB) signaling pathway [17]. Moreover, DUSP1 is also involved in the pro-apoptotic effects of the chemopreventive molecule resveratrol in prostate cancer cells [19]. In addition, it has been reported that DUSP1 inhibits cell migration, invasion, and metastasis in other cancer types [20,21,22,23,24]. However, despite all these studies showing DUSP1 as an apoptosis inducer in prostate cancer, the role of this phosphatase in cell migration and invasion in these kind of tumors remains largely unknown. Therefore, in this work, we aimed to investigate whether DUSP1 is involved in the motility of prostate cancer cells and whether this protein regulates the signaling pathways that control these processes. In brief, our results demonstrate that DUSP1 decreases Snail expression as well as cell migration and invasion in prostate tumor cells. Moreover, our data also support that DUSP1 regulates both processes, together with Snail expression, through the inactivation of c-Jun N-terminal kinase (JNK) and extracellular-signal-regulated kinase (ERK). Importantly, we also elucidate a new molecular pattern, which might be useful as a prognosis biomarker for prostate cancer monitoring. This molecular signature is characterized by an inverse correlation between DUSP1 and Snail levels with an additional activation of JNK and ERK pathways. Finally, our results show that expression of DUSP1 and Snail, as well as levels of active ERK and JNK correlate with time of progression and with exitus rate. In line with this, those patients with high DUSP1 expression, low JNK and ERK activities, and low Snail expression exhibit a longer time until they reach metastatic disease, a better outcome, and a lower exitus rate than those with the opposite expression pattern (DUSP1_low_/activated JNK_high_/activated ERK_high_/Snail_high_). Importantly, we consider that our findings suggest new opportunities to improve current strategies for the diagnosis and treatment of prostate cancer.

## 2. Materials and Methods

### 2.1. Cell Lines, Inhibitors, Plasmids, Cell Transfection and Luciferase Assay

DU145 and PC3 androgen-independent prostate cancer cells were purchased from the American Tissue Culture Collection (Manassas, UA, USA) and were cultured as recommended. The inhibitors were U0126 (Promega Biotech Ibérica, Madrid, Spain), SB203580, SP600125, and MG132 (Calbiochem, Merck Chemicals, Barcelona, Spain). The pCMV-DUSP1 and the Snail-Luc reporter plasmids were previously described [25,26]. For overexpression and siRNA experiments, cells were transiently transfected as previously described [19]. Luciferase assays were performed as in [27], being the luciferase levels normalized to those of renilla, and expressed as the induction over the controls.

### 2.2. Western Blot Analyses and Immunofluorescence Staining

Western blot analyses were performed as described in [27]. The antibodies were anti-DUSP1, anti-p38MAPK, anti-JNK1, and anti-ERK2 (Santa Cruz Biotechnology, Heidelberg, Germany); anti-phospho-p38MAPK (pp38MAPK), anti-phospho-ERK (pERK), and anti-Snail (Cell Signalling Technology, Izasa S.A., Barcelona, Spain); anti-phospho-pJNK (pJNK) (Promega Biotech Ibérica, Madrid, Spain); anti-Tubulin (Sigma Aldrich, Madrid, Spain); peroxidase-conjugated secondary antibodies (GE Healthcare Europe GMBH, Barcelona, Spain). Tubulin was utilized as a loading control for Western blotting analysis. Relative protein levels compared to tubulin were analyzed by Image J software and plotted.

Immunofluorescence staining was performed as previously described [28]. Briefly, cells cultured on coverslips were fixed, permeabilized, blocked and, after several washes, stained for Snail with the specific antibody, followed by the anti-rabbit Alexa Fluor^®^ 488 secondary antibody (BD Biosciences, Franklin Lakes, NJ, USA). Samples were mounted using ProLong^®^ Gold Antifade Mountant with DAPI (Invitrogen, Life Technologies, Carlsbad, CA, USA), and fluorescence visualization was performed by ICTS “NANBIOSIS”, more specifically by the Confocal Microscopy Service (Ciber in Bioengineering, Biomaterials & Nanomedicine (CIBER-BNN)) at the Alcalá University.

### 2.3. Cell Migration and Invasion Assays

Cell migration was examined by wound-healing assays. After transfection/treatment of cells, scratches were made using sterile 200 μL-pipette tips, and bright-field microphotographs were taken at different times. The percentages of cell migration were quantitated, by the ImageJ software, measuring the width of the cell-free zone immediately after making the scratch, and at different times after scratching. Migration velocities represented the average velocities at which the cells moved into the gap.

Cell invasion was examined in Matrigel-coated transwells (BD Biosciences, Franklin Lakes, NJ, USA) as previously described [29]. The number of cells loaded onto the surface of each Matrigel-coated transwell was 100,000 in DUSP1 overexpression and MAPK inhibitors experiments, and 50,000 in DUSP1 silencing experiments. Invaded cells were stained with crystal violet, and three different cell fields of each well were photographed under a phase contrast microscope (Nikon TS100). Changes in cell invasion were expressed as percentages of the corresponded controls.

### 2.4. Experimental Subjects and Immunohistochemistry of Prostate Tissues

Paraffin-embedded samples from patients diagnosed with BPH (*n* = 9) or PC (*n* = 35) were used (Table 1). Five-micron thick sections from samples were incubated overnight at room temperature with each primary antibody (anti-DUSP1 and anti-Snail1, clone G7 (Santa Cruz Biotechnology, Heidelberg, Germany); anti-pJNK (Promega, Promega Biotech Ibérica, Madrid, Spain); anti-pERK (Cell Signalling Technology, Izasa S.A., Barcelona, Spain)). Afterwards, samples were washed and sequentially incubated with the biotin free, peroxidase-detection system (polymer-based detection kit, MasVision^TM^, Master Diagnostica, Spain). Nuclei were stained with Caracci’s hematoxylin. Samples were dehydrated and mounted with DePex. The intensity of the immunostaining was evaluated by two independent observers who were blinded to patient clinical information through a system of subjective gradation. Immunostaining scores were ranged into four categories based on the staining pattern of the majority of tumor cells in the whole section, which were grouped into two main categories for statistical purposes (0–1: negative/low staining; 2–3: moderate/high staining).

### 2.5. Statistical Analyses

In the experiments with cell lines, all data were expressed as means ± SEM. Student’s *t* test was performed using the SSC-Stat software (V2.18, University of Reading, UK). In the immunohistochemistry assays, GraphPad Prisma 3.0 software was used for statistical purposes. Immunostaining score and clinical data were analyzed using one-way ANOVA and either the Bonferroni’s or Dunnet´s multiple comparison tests. The correlation among markers was analyzed using the Pearson´s test (95% confidence interval). Log-rank test and survival curves were used to determine the relationship among markers and time to clinical progression. The statistical significance of difference between groups was expressed by asterisks (* 0.01 < *p* < 0.05; ** 0.001 < *p* < 0.01; *** *p* < 0.001).

## 3. Results

### 3.1. DUSP1 Downregulates Snail Expression and Impairs Cell Migration and Invasion in Prostate Cancer Cells

**Figure 1:**
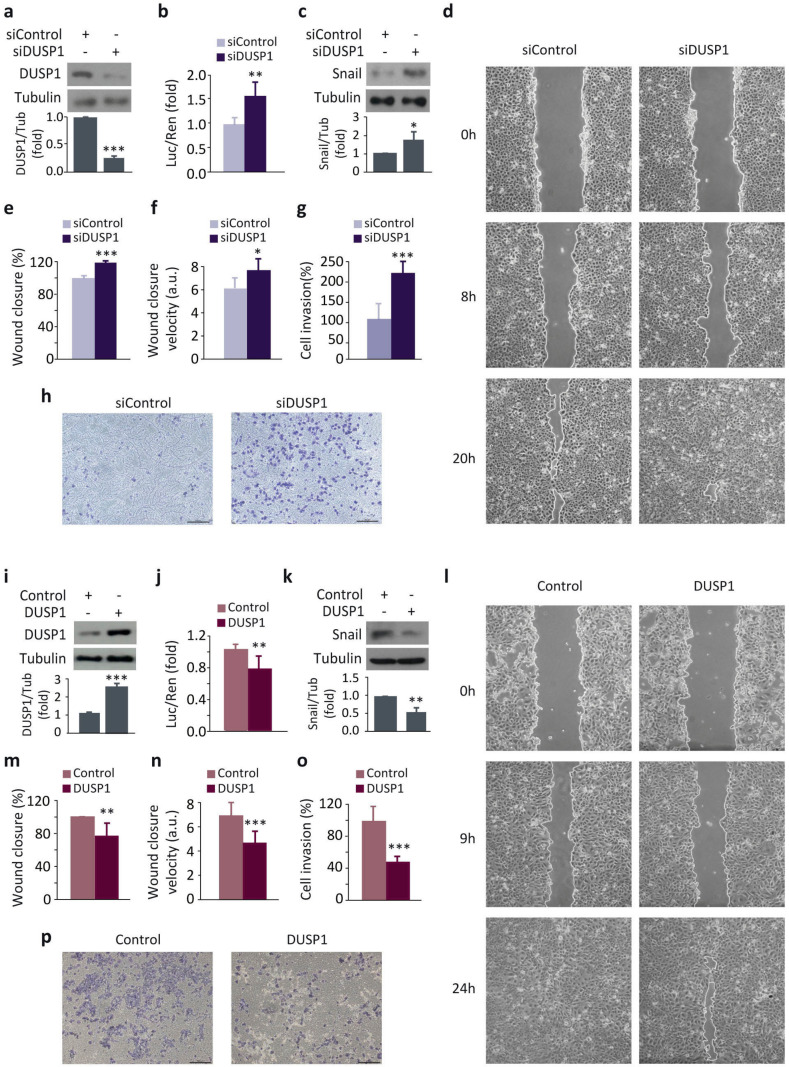
DUSP1 downregulates Snail expression and impairs cell migration and invasion in DU145 cells. (**a**) Cells were transfected for 48 h with the control siRNA (siControl) or the DUSP1 siRNA (siDUSP1) and expression levels of DUSP1 and Tubulin were determined by western blotting. (**b**) Cells were transfected for 48 h with the siControl or the siDUSP1 together with the Snail-Luc plasmid and luciferase activity was measured in cell extracts. (**c**) Cells were transfected as in *a* and expression levels of Snail and Tubulin were determined by western blotting. (**d**–**f**) Wound healing assay and measurement of wound closure area and velocity in cells transfected as in *a*. (**g**,**h**) Invasion capacity using transwell assays in cells transfected as in *a*. (**i**) Cells were transfected with a control vector (Control) or a vector encoding DUSP1 (DUSP1) and expression levels of DUSP1 and Tubulin were determined by western blotting. (**j**) Cells were transfected for 48 h with the Control or the DUSP1 vectors together with the Snail-Luc plasmid and luciferase activity was measured in cell extracts. (**k**) Cells were transfected with the Control or the DUSP1 vectors and expression levels of Snail and Tubulin were determined by western blotting. (**l**–**n**) Wound healing assay and measurement of wound closure area and velocity in cells transfected as in *i*. (**o**,**p**) Invasion capacity using transwell assays in cells transfected as in *i*. For all the results, data are shown as the mean ± SEM of at least three independent experiments. For migration and invasion assays, pictures are from one representative experiment of three with similar results. Student’s *t* test: * 0.01 < *p* < 0.05; ** 0.001 < *p* < 0.01; *** *p* < 0.001.

To study the role of DUSP1 in the migration and invasion of prostate cancer cells, we first analyzed the effect of DUSP1 knockdown on Snail expression in DU145 cells. DUSP1 silencing efficiency was tested by measuring its protein levels, observing a significant decrease in DUSP1-deficient cells (Figure 1a). The results showed an increase in Snail levels both at a transcriptional (Figure 1b) and at a protein level (Figure 1c). Consistently, DUSP1-deficient cells significantly displayed an enhanced capacity of both cell migration (Figure 1d–f) and invasion (Figure 1g,h). Conversely, cells overexpressing DUSP1 showed a significant increase in protein levels (Figure 1i), significantly reduced Snail expression levels (Figure 1j,k), were less migratory (Figure 1l–n), and displayed limited cell invasion (Figure 1o,p). Similar results were obtained from experiments performed in PC3 cells, thus ruling out the cell-type specific effects of this phosphatase (Figure S1 in Supplementary Materials). All these results indicate that DUSP1 downregulates Snail expression, which in turn results in a further decrease in migration and invasion of prostate cancer cells.

### 3.2. The Inhibition of JNK and ERK Downregulates Snail Expression, Cell Migration and Invasion

**Figure 2:**
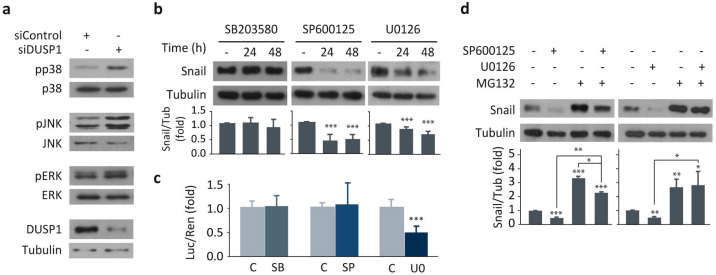
The inhibition of JNK and ERK downregulates Snail expression in DU145 cells. (**a**) Cells were transfected for 48 h with the siControl or the siDUSP1 and expression levels of DUSP1, phosphorylated MAPKs (pp38, pJNK, pERK), total MAPKs and Tubulin were determined by western blotting. (**b**) Cells were incubated at different times in the absence or presence of 1 μM SB203580 (SB), 10 μM SP600125 (SP) or 20 μM U0126 (U0), and expression levels of Snail and Tubulin were determined by western blotting. (**c**) Cells were transfected with the Snail-Luc plasmid, incubated for 48 h as in *b* and luciferase activity was assayed in cell extracts. (**d**) Cells were incubated for 48 h with 10 μM SP600125 or 20 μM U0126, treated in the absence or presence of 10 μM MG132 for the last 4 h and expression levels of Snail and Tubulin were determined by western blotting. For all the results, data are shown as the mean ± SEM of at least three independent experiments. Student’s t test: * 0.01 < *p* < 0.05; ** 0.001 < *p* < 0.01; *** *p* < 0.001.

Given that DUSP1 is able to dephosphorylate and inhibit different MAPK signaling pathways, we next investigated which of them were involved in the effects of this phosphatase on Snail expression, cell migration, and invasion in DU145 cells. Our results confirmed that p38MAPK, JNK, and ERK were targets of this phosphatase, since the abrogation of its expression activated these three MAPKs (Figure 2a). In addition, the inhibitory effect of DUSP1 on MAPK’s activities was confirmed by monitoring the levels of their phosphorylated forms in cells overexpressing this phosphatase (data not shown).

Further analysis of Snail expression after inactivation of these MAPKs was performed upon treatment of cells with specific inhibitors. The efficiency of selective inhibition of MAPK activity by SB203580 (p38MAPK inhibitor), SP600125 (JNK inhibitor), or U0126 (MEK inhibitor) was confirmed by measuring MAPK phosphorylation levels in cells incubated with these compounds (Figure S2 in Supplementary Materials). Moreover, the inhibition of these MAPKs differently affected cell proliferation and survival [17] (unpublished results). Regarding Snail expression, the inhibition of p38MAPK with SB203580 did not affect Snail expression (Figure 2b,c). In contrast, treatment with either SP600125 or U0126 achieved a significant reduction in Snail levels (Figure 2b), although only ERK inhibition exerted its effects at a transcriptional level (Figure 2c). Moreover, the effect of JNK and ERK inhibition on Snail proteasomal degradation was assessed, and the analysis of these data revealed that the reduction in Snail levels achieved by SP600125 or U0126 was reversed by the inhibitor MG132 (Figure 2d), suggesting that Snail regulation by JNK or ERK pathways is proteasome dependent. 

**Figure 3:**
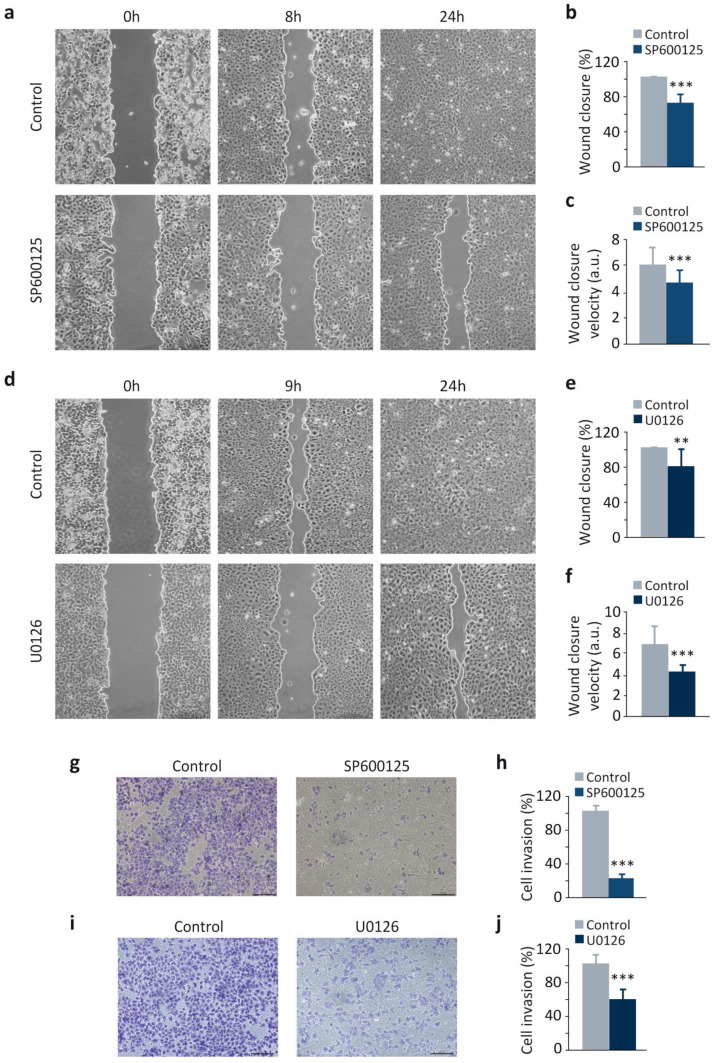
The inhibition of JNK and ERK decreases migration and invasion in DU145 cells. (**a**–**f**) Wound healing assay and measurement of wound closure area and velocity in cells incubated for 48 h with 10 μM SP600125 (**a**–**c**) or 20 μM U0126 (**d**–**f**). (**g**–**j**) Invasion capacity using transwell assays in cells incubated as above. For all the results, data are shown as the mean ± SEM of at least three independent experiments. Pictures are from one representative experiment of three with similar results. Student’s *t* test: ** 0.001 < *p* < 0.01; *** *p* < 0.001.

Additionally, both JNK and ERK inhibition reduced cell migration (Figure 3a–f) and invasion (Figure 3g–j), mimicking the results obtained following DUSP1 overexpression (Figure 1j–n). In contrast, p38MAPK inhibition did not affect cell migration (Figure S3 in Supplementary Materials), suggesting that this kinase is supporting other processes in prostate cancer progression. All these results, together with those showed in Figure 1, demonstrate that both pharmacological inhibition of JNK or ERK and DUSP1 overexpression exert similar effects on Snail expression, cell migration, and invasion, suggesting that this phosphatase regulates these processes by specifically targeting these two pathways.

### 3.3. Snail Subcellular Location Is Regulated by the Phosphatase DUSP1 and JNK and ERK Signaling Pathways

**Figure 4:**
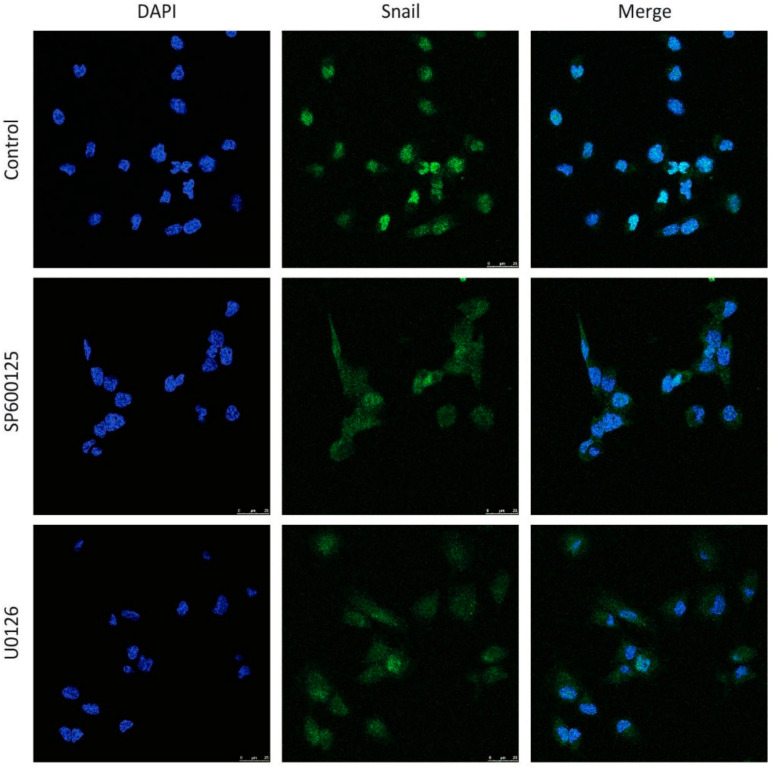
Snail subcellular location is regulated by the JNK and ERK signaling pathways. DU145 cells were incubated for 48 h with 10 μM SP600125 or 20 μM U0126 and Snail subcellular location was determined by immunofluorescence as described in Material and methods. DAPI was used to identify the nuclei. Pictures are from one representative experiment of three with similar results.

One of the most common molecular mechanisms by which Snail expression is downregulated involves its nuclear export to the cytoplasm and its subsequent proteasomal degradation. Since we demonstrated that JNK and ERK inhibition decreased Snail expression by affecting its proteasomal degradation (Figure 2d), we next analyzed Snail location upon treatment with the specific MAPKs inhibitors. As expected, our results showed that SP600125 and U0126 induced a more diffuse location of Snail with an increase in the cytosolic compartment (Figure 4).

**Figure 5:**
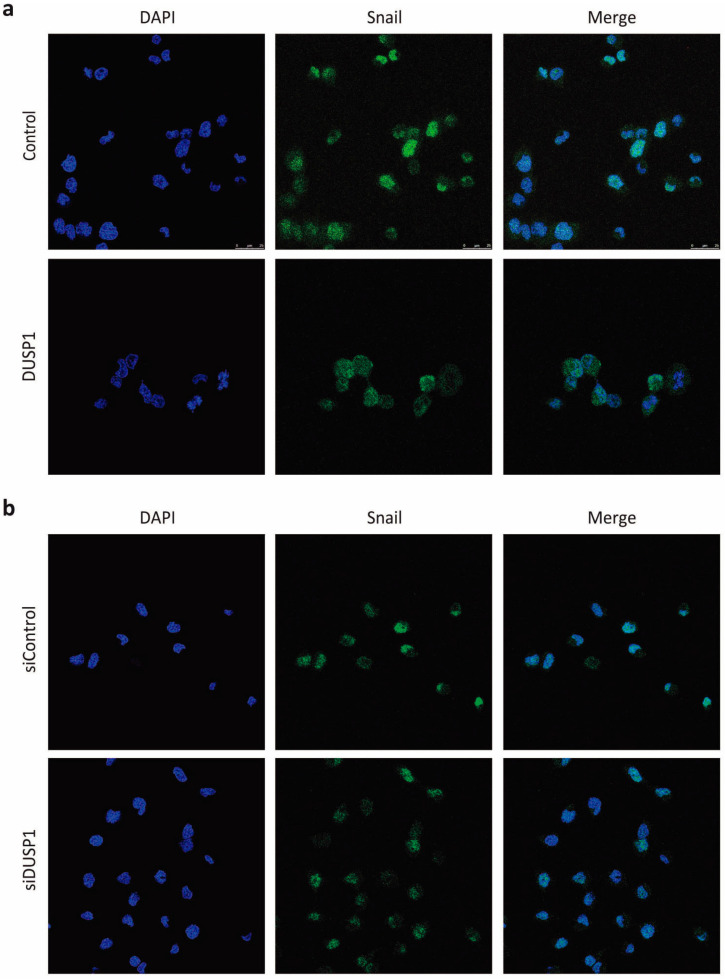
Snail subcellular location is regulated by the phosphatase DUSP1. (**a**) DU145 cells were transfected for 48 h with the Control or the DUSP1 vectors. (**b**) Cells were transfected for 48 h with the siControl or the siDUSP1. In both set of experiments, Snail subcellular location was determined by immunofluorescence as described in Material and methods. DAPI was used to identify the nuclei. Pictures are from one representative experiment of three with similar results.

Consistently, DUSP1 overexpression also induced a predominantly cytosolic location of Snail, while DUSP1 knockdown maintained this transcription factor in the nucleus (Figure 5). These results reveal that both DUSP1 overexpression and JNK or ERK inhibition induce the export of Snail from the nucleus to the cytoplasm; hence, these data strengthen our hypothesis that this phosphatase exerts its effects on Snail subcellular location through the downregulation of these MAPKs.

### 3.4. JNK and ERK Cooperatively Regulate Snail Expression, Cell Migration and Invasion

**Figure 6:**
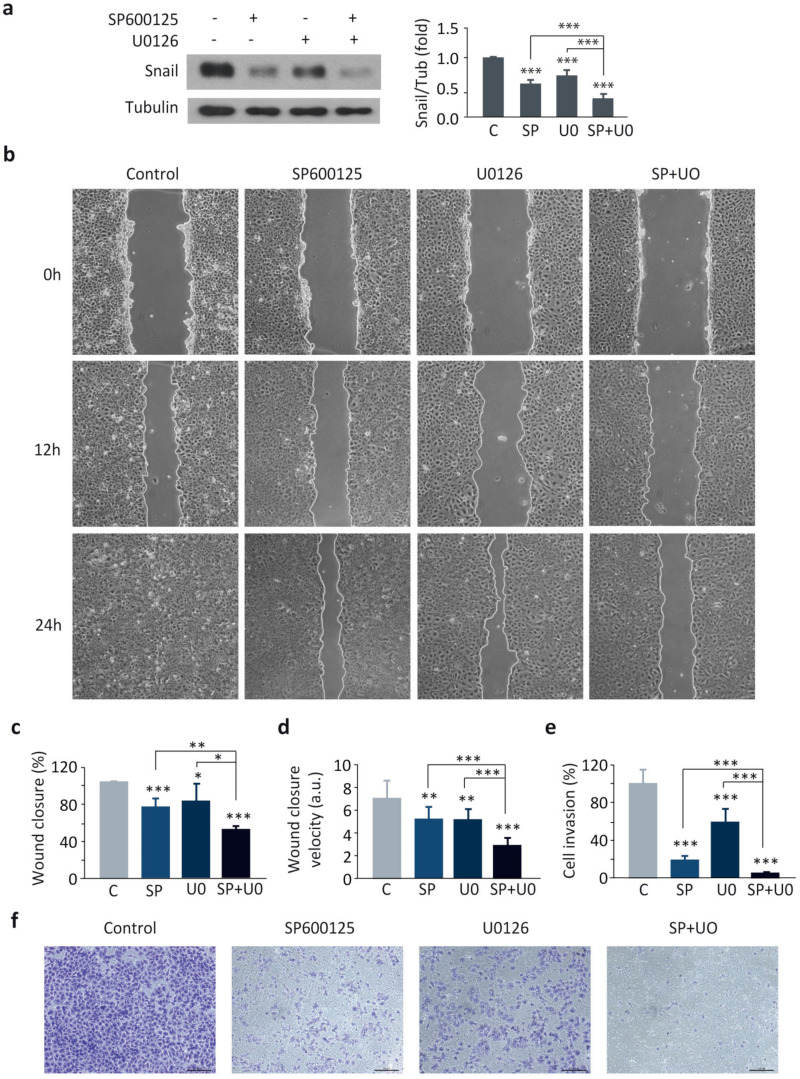
JNK and ERK cooperatively regulate Snail expression, cell migration and invasion in DU145 cells. Cells were incubated in the absence (C) or presence of 10 μM SP600125 (SP, 24 h) and 20 μM U0126 (U0, 48 h). (**a**) Expression levels of Snail and Tubulin were determined by western blotting. (**b**–**d**) Wound healing assay and measurement of wound closure area and velocity. (**e**,**f**) Invasion capacity using transwell assays. For all the results, data are shown as the mean ± SEM of at least three independent experiments. For migration and invasion assays, pictures are from one representative experiment of three with similar results. Student’s *t* test: * 0.01 < *p* < 0.05; ** 0.001 < *p* < 0.01; *** *p* < 0.001.

Given that DUSP1 impaired the activity of JNK and ERK (Figure 2a), and that the individual inhibition of these MAPKs downregulated Snail expression (Figure 2b), as well as cell migration and invasion (Figure 3), we further studied whether these MAPKs cooperated in the regulation of these events in our prostate cancer cells. Interestingly, the combination of SP600125 and U0126 significantly achieved a higher reduction in Snail expression than the single treatments in DU145 cells (Figure 6a).

Notably, cells treated with SP600125 plus U0126 were even less migratory (Figure 6b–d) and displayed less invasion capacity (Figure 6e,f) compared to cells treated with the single agents. To further strengthen these results, we extended our study, performing similar experiments in PC3 cells. As expected, our results showed that JNK and ERK cooperatively regulated Snail expression and cell migration also in these cells (Figure S4 in Supplementary Materials). All these results indicate that the dual inhibition of JNK and ERK pathways in prostate cancer cells is more effective in decreasing Snail expression, cell migration, and invasion than blocking each pathway independently. Altogether, these results suggest once again that DUSP1 regulates these events through a dual inhibition of both JNK and ERK pathways.

### 3.5. DUSP1 Expression Inversely Correlates with Snail Levels and Activated JNK and ERK in Human Prostate Samples

**Figure 7:**
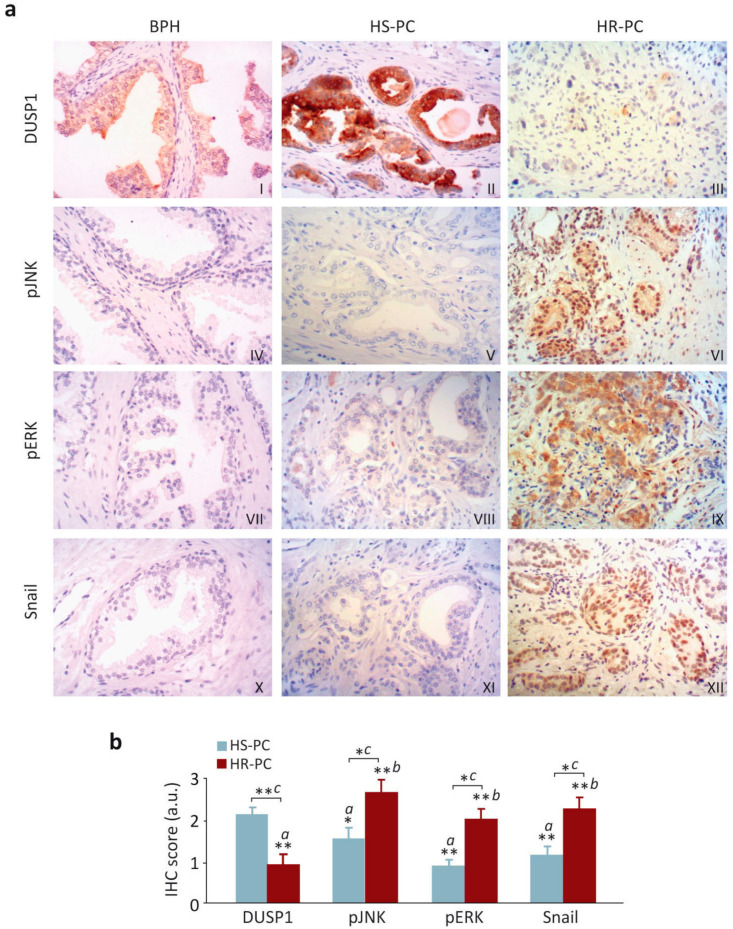
DUSP1 expression inversely correlates with Snail levels and activated JNK and ERK in human prostate samples. (**a**) Immunohistochemical analysis of expression levels of DUSP1 (I–III), phosphorylated JNK (pJNK, IV–VI), phosphorylated ERK (pERK, VII–IX) and Snail (X–XII) from human prostate cancer samples. Micrographs were taken at 200× magnification and show serial sections from the same gland stained with each one of the four used antibodies. (**b**) Immunohistochemical score for DUSP1, pJNK, pERK and Snail in samples from HS-PC and HR-PC. The statistical analysis was performed with One-way ANOVA and Dunnet´s multiple comparison test, and asterisks show the statistical significance of differences between the groups (*a:* comparison with DUSP1 from HS-PC samples; *b:* comparison with DUSP1 from HR-PC samples; *c:* HS-PC vs HR-PC for each marker), * 0.01 < *p* < 0.05; ** 0.001 < *p* < 0.01.

To investigate whether our results obtained from the experiments performed with the cell lines were clinically relevant, we next analyzed the expression levels of DUSP1 and Snail in a series of samples from patients with BPH, HS-PC, and HR-PC (Table 1). Prostatic glands from BPH samples showed a high expression of DUSP1 (Figure 7a-I) and a weak expression of Snail (Figure 7a-X). In prostate cancer samples, DUSP1 expression was high in HS-PC (Figure 7a-II), whereas low or no signal for Snail was detected (Figure 7a-XI). Conversely, HR-PC samples showed a weak or even undetectable DUSP1 expression (Figure 7a-III) but a moderate to strong signal for Snail (Figure 7a-XII). Consequently, the immunohistochemical analyses demonstrated an inverse correlation between DUSP1 and Snail, with a DUSP1_high_/Snail_low_ pattern in both BPH and HS-PC samples, and a DUSP1_low_/Snail_high_ pattern in HR-PC samples. Importantly, results from the Pearson´s Test confirmed the inverse correlation between DUSP1 and Snail expression (Figure 7b).

Since our data in prostate cancer cells revealed that DUSP1 inhibits JNK and ERK (Figure 2a) and these MAPKs negatively regulated Snail expression (Figure 2b–d), we also analyzed the levels of activated JNK and ERK (pJNK and pERK) in patient samples. Accordingly, our results indicated that the levels of active JNK and ERK were low in BPH samples (Figure 7a-IV,VII). Moreover, an inverse correlation was also detected for PC samples, with a DUSP1_high_/pJNK_low_ /pERK_low_ pattern in samples from HS-PC patients (Figure 7a-II,V,VIII) and a DUSP1_low_/pJNK_high_ /pERK_high_ pattern in HR-PC samples (Figure 7a-III,VI,IX). As in previous results, the Pearson´s Test confirmed these inverse correlations (Figure 7b).

In all cases, subcellular localization for DUSP1 and pERK was mainly cytosolic, while Snail was located in the cell nucleus. Regarding pJNK subcellular expression, it was predominantly nuclear, although a mild-to-moderate signal for this marker was also observed in cytosol (Figure S5 in Supplementary Materials). Moreover, a compilation of different IHC images for each marker can be observed in Figure S6 in Supplementary Materials.

### 3.6. The Relationship of DUSP1 and Snail Levels and JNK and ERK Activities Are Associated with Disease Progression and Clinical Outcome in Patients with Prostate Cancer

**Figure 8:**
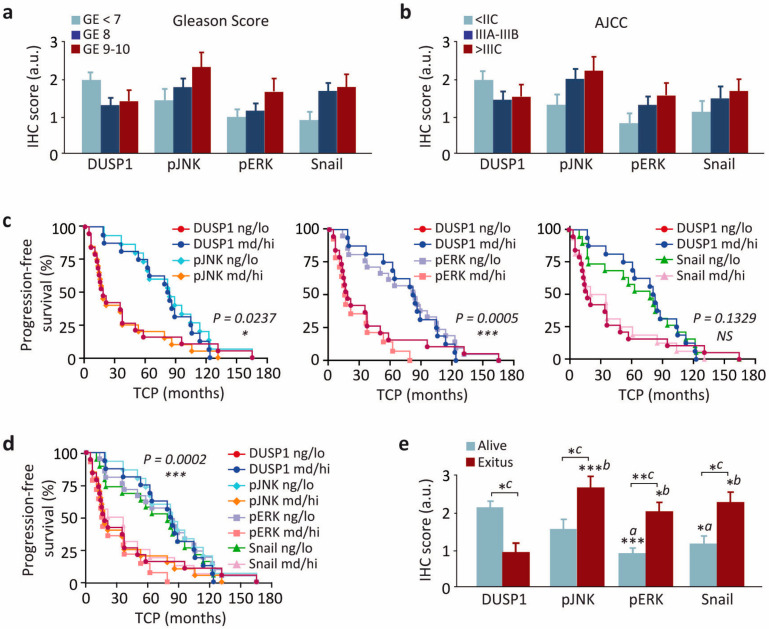
The relationship of DUSP1 and Snail levels and JNK and ERK activities are associated with disease progression and clinical outcome in patients with prostate cancer. (**a**,**b**) Immunohistochemical score for DUSP1, phosphorylated JNK and ERK (pJNK and pERK) and Snail in samples ranged into three categories based on their Gleason Score (**a**) or AJCC group staging at diagnosis (**b**). (**c**) Progression-free survival of patients showing immunohistochemical score for DUSP1/pJNK, DUSP1/pERK or DUSP1/Snail. Samples were ranged into two categories based on the staining pattern of the majority of tumor cells in the whole section (negative/low (ng/lo); moderate/high (md/hi)). (**d**) Progression-free survival of patients showing immunohistochemical score for DUSP1/pJNK/pERK/Snail. Samples were ranged into two categories as described in *c*. (**e**) Immunohistochemical score for DUSP1, pJNK, pERK and Snail in samples from patients either alive or dead. The statistical analysis was performed with One-way ANOVA and Dunnet´s multiple comparison test, and asterisks show the statistical significance of differences between the groups (*a:* comparison with DUSP1 from HS-PC samples; *b:* comparison with DUSP1 from HR-PC samples; *c:* HS-PC vs HR-PC for each marker). *TCP, Time to clinical progression*, * 0.01 < *p* < 0.05; ** 0.001 < *p* < 0.01; *** *p* < 0.001.

Since we observed a differential expression of DUSP1, Snail, and the active forms of JNK and ERK in samples from prostate cancer patients at different stages, we next studied the interrelation between the levels of these proteins and some of the most important clinical parameters. Firstly, we analyzed the correlation of expression patterns of DUSP1, Snail, and activated JNK and ERK with either Gleason score (Figure 8a) or American Joint Committee on Cancer (AJCC) group staging at diagnosis [30] (Figure 8b), and no correlation was observed in any of these cases. In contrast, we did observe a significant correlation when we compared the levels of DUSP1, Snail, and activated JNK and ERK with both the disease progression and the clinical outcome (Figure 8c–e). Thus, shorter intervals to clinical progression were related with lower DUSP1 expression and higher levels of activated JNK (*log-rank*, *p* = 0.0237) and ERK (*log-rank*, *p* = 0.0005) (Figure 8c), although we did not observe correlation of time to clinical progression with lower DUSP1 expression and higher levels of Snail (Figure 8c). Despite this, the combined pattern DUSP1_low_/pJNK_high_/pERK_high_/Snail_high_ was strongly related with overall time to clinical progression (*log-rank*, *p* = 0.0002) (Figure 8d). More importantly, our data also evidenced a significant relationship between the expression pattern of these proteins and exitus (Figure 8e). Indeed, the median overall survival of patients with the combined pattern DUSP1_low_/pJNK_high_/pERK_high_/Snail_high_ was 29 months, compared to 79 months in patients with DUSP1_high_/pJNK_low_/pERK_low_/Snail_low_.

Collectively, all the results in human prostate samples reveal the existence of an inverse correlation between DUSP1 expression and the levels of Snail and activated JNK and ERK (negative correlation at Pearson´s test, *p* < 0.001), supporting our experiments in prostate cancer cells which demonstrate that DUSP1 downregulates Snail expression. In addition, our results indicate that low levels of DUSP1 and high levels of pJNK (*p* < 0.02) and pERK (*p* < 0.0005), but not Snail (*p* > 0.05), are related to shorter intervals to clinical progression. Finally, and more interestingly, we evidence that the levels of all proteins tested are related to clinical outcome, suggesting that the ratio between the expression of DUSP1, Snail, and activated JNK and ERK is an important marker for diagnostic purposes in prostate cancer.

## 4. Discussion

DUSP1 expression has been previously related to different stages of human prostate carcinomas. In line with this, the expression of this phosphatase is high in BPH and HS-PC, but it is lost in later stages, such as HR-PC [17]. Furthermore, DUSP1 overexpression in androgen-independent prostate cancer cells induces apoptosis through both p38MAPK and NF-kB dependent mechanisms [17]. Here, we show for the first time that this phosphatase plays an additional anti-tumorigenic role in prostate cancer cells, since it decreases the expression levels of the EMT master regulator, Snail, and inhibits cell migration and invasion through the inactivation of JNK and ERK. Interestingly, we also demonstrate a correlation between the expression levels of DUSP1 and Snail and the activity of JNK and ERK in samples from prostate cancer patients, discovering a novel approach to predict the prognosis and outcome of this disease.

Previous studies have shown that the overexpression of Snail in prostate cancer cells is associated with an increased cell migration and invasion, while its silencing induces a decrease in these processes [31]. In agreement with this, here, we demonstrate that DUSP1 downregulates Snail expression and inhibits migration and invasion in prostate cancer cells. Our data are similar to those observed in different types of tumors, in which DUSP1 suppresses cell migration, cell invasion, metastasis, and/or angiogenesis by inhibiting either ERK [21,23], JNK [22,24], or p38MAPK [20]. Consistently with DUSP1 effects on MAPK activity, the ERK pathway is one of the major oncogenic signals in human cancers because its activation leads to an increase in proliferation, invasion, and metastasis [32]. Particularly in prostate cancer, the ERK pathway is often hyperactivated [33], acts as an inducer of cell migration and invasion [34,35] through a Snail-mediated mechanism [36], and is involved in the effects of different molecules on these processes [37,38,39]. In addition, the JNK pathway has also been described to be important as a pro-tumorigenic signal through Snail regulation in different tumors [40,41,42]. Regarding prostate cancer, it has been previously described that JNK activity is related to elevated cell migration and invasion [43] and controls tumor growth in DU145 prostate carcinoma xenografts [44], although the involvement of Snail in these processes is still unknown. Our results are in agreement with all these data, since we demonstrate that the effects of DUSP1 on Snail levels, cell migration, and cell invasion are similar to those observed upon specific inhibition of the ERK and JNK pathways. By contrast, our findings evidence that p38MAPK is not involved in the regulation of these processes by DUSP1. Although several reports have showed that this kinase promotes cancer by enhancing migration in tumor cells [45], we demonstrate that the pro-tumorigenic role of p38MAPK in prostate cancer is more related to its effects on cell apoptosis [17] than to those involved in cell migration and invasion. Overall, all these data suggest that the role that DUSP1 plays as a tumor suppressor in prostate cancer is complex and depends on the specific inactivation of one or the other MAPK, which ultimately controls either cell apoptosis, or cell migration and invasion.

The regulatory mechanisms that control the cellular levels of Snail are very complex and involve changes at the transcriptional level or post-translational modifications, which affect its location in the cell nucleus and/or cytosol, as well as its susceptibility to degradation [16]. Here, we show for the first time that DUSP1 expression regulates the transcription of Snail. Moreover, only the concomitant ERK inhibition affects Snail expression at this level, while JNK controls it exclusively at protein level. Similar data in other cancer cell contexts have shown that the activation of Snail transcription requires an active ERK pathway [46], whereas no data on JNK involvement in this process have been reported. Regarding the regulation of Snail at a protein level, several mechanisms control the migration and invasion of prostate cancer cells by modulating the location and stability of this transcription factor. In this regard, one of the most common regulatory mechanisms is the phosphorylation of Snail by glycogen synthase kinase 3 beta (GSK-3β), which induces its nuclear export to cytosol and marks this protein for degradation in prostate cancer [47,48,49]. Interestingly, active ERK phosphorylates and inhibits GSK-3β, maintaining Snail in an active non-phosphorylated state and located at the cell nucleus [50]. Thus, the location of Snail in the cytosol promoted by DUSP1-dependent ERK inactivation is a possible mechanism that explains the decrease of Snail levels following DUSP1 overexpression. However, other regulatory mechanisms of Snail expression, independent of GSK-3β, have been previously identified in different tumors. For example, in hepatocarcinoma and breast cancer cells, the JNK pathway upregulates the lysil oxidase-2 (LOXL-2) [51], which oxidizes Snail, preventing its phosphorylation by GSK-3β [52]. In prostate cancer cells, elevated levels of LOXL-2 have been detected [53], supporting the possible involvement of this protein in the effects of the JNK pathway on the prostatic carcinogenesis. Alternatively, our group has previously shown that Snail expression is regulated by ERK and an autocrine loop involving transforming growth factor beta (TGFβ)/Src/focal adhesion kinase (FAK) complex in thyroid cancer cells [28]. Similarly, other authors have demonstrated that FAK activation induces Snail expression and enhances mesothelial cell migration, promoting peritoneal metastasis from ovarian cancer [54]. Moreover, the JNK pathway activates migration by inducing the phosphorylation of paxillin, which is an adaptor protein related to FAK activation in different cancer cells [55,56]. In this regard, DUSP22, a member of the DUSP1 family which reduces JNK activation, negatively regulates cell migration through FAK dephosphorylation and inactivation in lung cancer cells [57]. Given that FAK and paxillin expression is elevated in prostate cancer and both proteins are associated with tumor progression, lymph node metastasis, and/or shortened survival [58,59], it is also plausible that in our cancer model, the paxillin/FAK pathway could contribute to the regulation of Snail expression by ERK and JNK. However, due to the difference between ERK- and JNK-dependent mechanisms, further research is required to investigate the molecular mechanisms underlying Snail regulation by these kinases.

Interestingly, we also demonstrate in this work the existence of an inverse correlation between DUSP1 and Snail expression levels in patients with different stages of prostate cancer. Importantly, in BPH and HS-PC samples, high levels of this phosphatase and low or none Snail expression were detected, while in HR-PC samples, either low or no DUSP1 expression and high Snail levels were observed. In agreement with our results, an increase in Snail expression has been related to disease progression, since there are higher levels of this protein in bone metastasis from prostate cancer compared to BPH samples [13,14,15]. Furthermore, other studies indicate that 66% of patients with prostatic adenocarcinoma show elevated Snail levels [60]. Here, we add new related information, demonstrating for the first time that Snail expression in patient samples is inversely correlated with DUSP1 levels and directly correlated with activated ERK and JNK pathways. In addition, the increase of active ERK in samples of HR-PC compared to those of HS-PC or BPH observed in our study is coincident with previous works. Accordingly, higher levels of phosphorylated ERK are found in samples obtained from tumors in advanced or metastatic phase, with respect to more localized tumors or BPH samples [61,62]. However, to our knowledge, this is the first study showing that the level of activated JNK is increased in prostate tumors with a more invasive phenotype, as previously seen in breast and urothelial carcinomas [63,64]. All these data obtained from the experiments carried out with patient samples confirm the results derived from our experimental cell line models and suggest that DUSP1 regulates prostate tumor progression by controlling Snail expression through ERK and JNK inactivation.

The presence of Snail has been strongly associated in prostate tumors with a high Gleason score [13,60] but not with other parameters such as the risk of recurrence or the Stage T [13]. In fact, no significant differences have been previously found in Snail expression in non-metastatic, non-recurrent cancer, recurrent cancer, or metastatic cancer at the time of diagnosis, suggesting that increased Snail expression is a relatively early event in the progress of the disease [13]. Most of the samples we analyzed in this study were locally advanced cancers. In fact, just one of our samples was graded as Gleason 6. Intermediate-risk Gleason grade 7 is usually considered as an individual group between grade 6 or lower and grade 8 or higher. Previous studies focused on the differences among the lower and the higher grades, but usually, no significant differences among grade 7 and higher grades were reported. When we correlated the expression of DUSP1, Snail, and activated ERK and JNK to clinical information, we found that their expression patterns did not correlate with either Gleason score or AJCC group staging at diagnosis. However, our results demonstrate that the pattern DUSP1_low_/pJNK_high_/pERK_high_/Snail_high_ is closely related with a worse survival. This observation is in agreement with previous data showing that DUSP1 expression correlates with better prognosis in glioblastoma [22] and with other studies where the association of Snail expression with a worse prognosis in prostate cancer was reported [13]. Therefore, since low DUSP1 expression and high levels of Snail and activated JNK and ERK are positively associated with final outcome (death), we can conclude that besides the overall immunohistochemical profile, high levels of Snail might be considered an independent indicator of bad prognosis that is predictive for worst outcome independently of time to progression. Moreover, since the expression pattern DUSP1_high_/pJNK_low_/pERK_low_/Snail_low_ is associated with an overall extended survival of patients and decreased cell migration and invasion, our results suggest that therapies based on DUSP1 induction combined with ERK and/or JNK inhibition may be promising in the treatment of metastatic prostate cancer.

## 5. Conclusions

**Figure 9:**
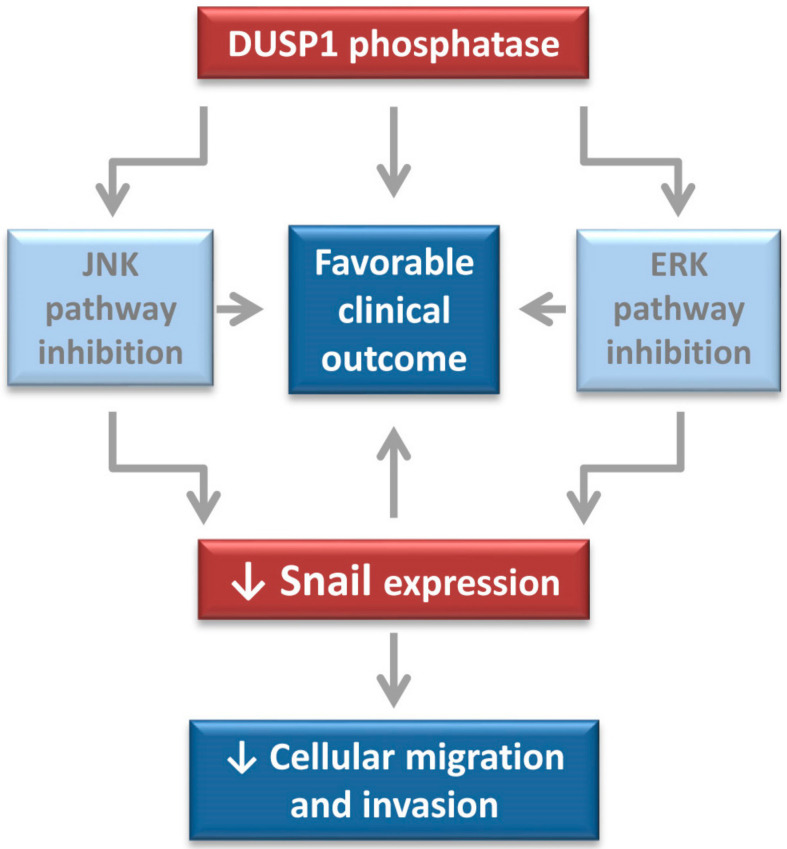
The phosphatase DUSP1 regulates metastasis-associated events in prostate cancer. This study demonstrate that DUSP1 overexpression downregulates Snail levels and decreases cell migration and invasion. Moreover, DUSP1 inactivates ERK and JNK pathways, whose inhibition exert similar effects on Snail expression, cell migration and invasion than overexpression of the phosphatase. In addition, JNK and ERK cooperate to regulate Snail expression, cell migration and invasion through different mechanisms. Finally, in clinical samples, the expression pattern DUSP1_high_/activeJNK_low_/activeERK_low_/Snail_low_ is associated with overall extended survival of patients and may serve as potential biomarker for identifying patients with favorable clinical outcome.

Our study provides new insights about the molecular mechanisms underlying the effects of the phosphatase DUSP1 on metastasis-associated events in prostate cancer (Figure 9). In summary, our experiments show that the overexpression of this phosphatase downregulates Snail levels and decreases cell migration and invasion, whereas DUSP1 silencing shows opposite effects. Moreover, we demonstrate that DUSP1 inactivates JNK and ERK pathways. Interestingly, the inhibition of these two kinases leads to similar effects on Snail expression, cell migration, and invasion to those observed following the overexpression of this phosphatase. In addition, JNK and ERK cooperate to regulate Snail levels, cell migration, and invasion through different mechanisms. Strikingly, we also demonstrate in human prostate tissue samples an inverse correlation between DUSP1 levels and both active JNK and ERK, as well as Snail expression. Thus, we show that the expression pattern DUSP1_high_/pJNK_low_/pERK_low_/Snail_low_ is associated with the overall extended survival of patients. Based on all these data, we conclude that the ratio between the expression levels of DUSP1 and Snail could be an important biomarker for diagnostic purposes in prostate cancer, as they may serve for identifying patients at risk for an unfavorable clinical outcome. In addition, our results strongly suggest that the induction of DUSP1 or the inhibition of ERK and JNK pathways could be useful as a therapeutic approach to treat prostate cancer.

## Figures and Tables

**Table 1:** Clinical data of prostate cancer patients (*n* = 35).

CLINICAL DATA	*n*
Age (median = 65)	
<65	15
≥65	20
Gleason grade	
≤7	13
>7	22
Invasivity (T)	
T1	5
T2	11
T3	15
T4	4
Metastatic disease at diagnostic (M)	
M0	31
M1	4
Response to androgen blockade	
Hormone-responsive (HS)	20
Hormone-refractory (HR)	15
**OUTCOME**	*n*
Alive	22
Exitus	13
**PROGRESSION**	months
Median survival	16
Time to biochemical progression	15
Time to clinical progression	50

## Data Availability

No new data were created or analyzed in this study. Data sharing is not applicable to this article.

## Supplementary Materials

The following are available online at https://www.mdpi.com/2072-6694/13/5/1158/s1, Figure S1: The phosphatase DUSP1 regulates Snail expression and migration in PC3 cells; Figure S2: The MAPKs selective inhibitors reduce MAPK activation; Figure S3: The inhibition of p38MAPK does not affect cell migration; Figure S4: JNK and ERK cooperatively regulate Snail expression and migration in PC3 cells; Figure S5: Immunohistochemical analysis showing details of subcellular localization of DUSP1, pERK, pJNK and Snail levels in samples from BPH, HS-PC and HR-PC selected from Figure 7; Figure S6: Immunohistochemical analysis of expression levels of DUSP1, pERK, pJNK and Snail in samples from three different patients diagnosed with BPH, HS-PC or HR-PC.
