# Supplementary material for: Downregulation of Snail by DUSP1 Impairs Cell Migration and Invasion through the Inactivation of JNK and ERK and Is Useful as a Predictive Factor in the Prognosis of Prostate Cancer
Source: Cancers (Basel). 2021 Mar 8;13(5):1158. doi: 10.3390/cancers13051158 (PMC7962644; doi:10.3390/cancers13051158)
Supplement: Supplementary file 1 [file cancers-13-01158-s001.pdf]

cancers

MDPI

Article

Supplementary Material: Downregulation of Snail by DUSP1 Impairs Cell Migration and Invasion through the Inactivation of JNK and ERK and Is Useful as a Predictive Factor in the Prognosis of Prostate Cancer

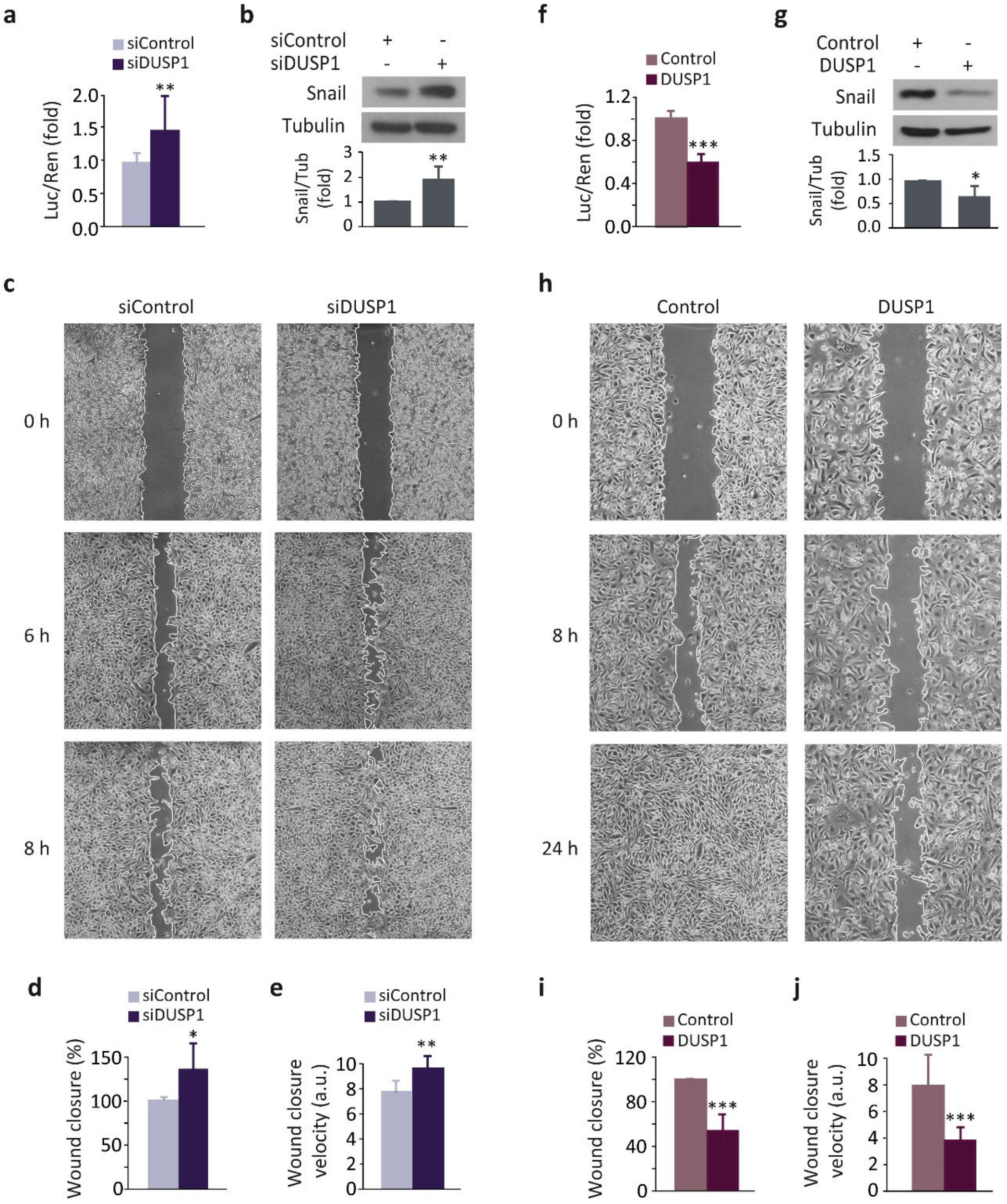

Figure S1. The phosphatase DUSP1 regulates Snail expression and migration in PC3 cells. (**a**) Cells were transfected for 48 h with the siControl or the siDUSP1 together with the Snail-Luc plasmid and cell extracts were assayed for

luciferase activity. (**b**) Cells were transfected for 48 h with the siControl or the siDUSP1 and expression levels of Snail and Tubulin were determined by western blotting. (**c–e**) Wound healing assay and measurement of wound closure area and velocity in cells transfected as in *b*. (**f**) Cells were transfected for 48 h with the Control or the DUSP1 vectors together with the Snail-Luc plasmid and luciferase activity was measured in cell extracts. (**g**) Cells were transfected for 48 h with the Control or the DUSP1 vectors and expression levels of Snail and Tubulin were determined by western blotting. (**h–j**) Wound healing assay and measurement of wound closure area and velocity in cells transfected as in *g*. For all the results, data are shown as the mean ± SEM of three independent experiments. For migration assays, pictures are from one representative experiment of three with similar results. Student's t test: * 0.01 < p < 0.05; ** 0.001 < p < 0.01; *** p < 0.001.

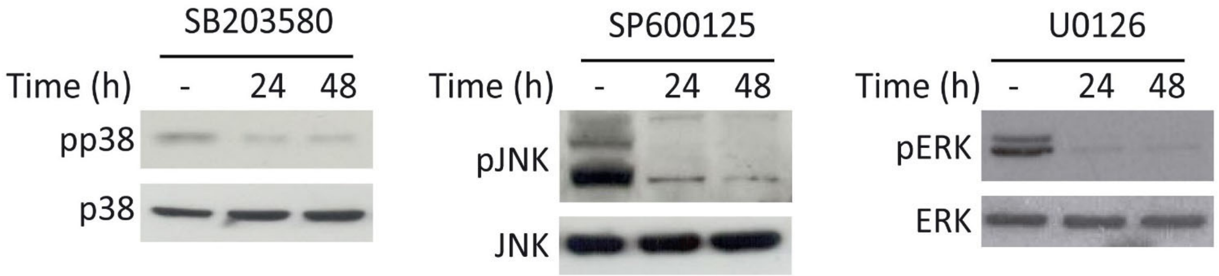

Figure S2. The MAPKs selective inhibitors reduce MAPK activation. DU145 cells were incubated at different times in the absence or presence of 1 μM SB203580, 10 μM SP600125 or 20 μM U0126 and expression levels of phosphorylated MAPKs (pp38, pJNK, pERK) and total MAPKs were determined by western blotting.

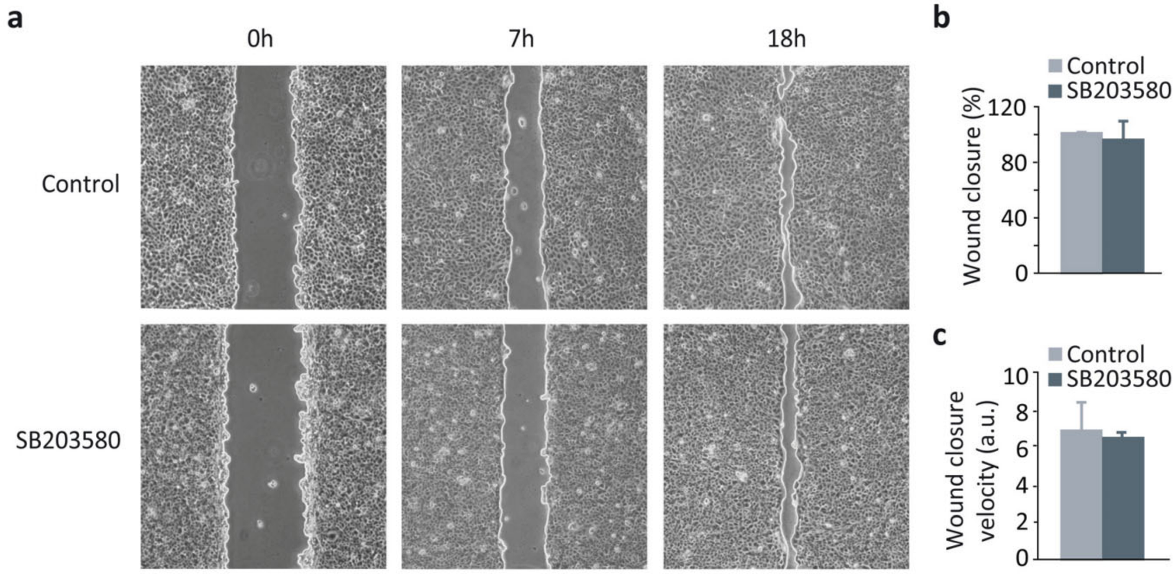

Figure S3. The inhibition of p38MAPK does not affect cell migration. DU145 cells were incubated for 48 h in the absence or presence of 1 μM SB203580. (**a**) Wound healing assay. (**b,c**) Measurement of wound closure area and velocity. Data are shown as the mean ± SEM of three independent experiments. The pictures are from one representative experiment of three with similar results.

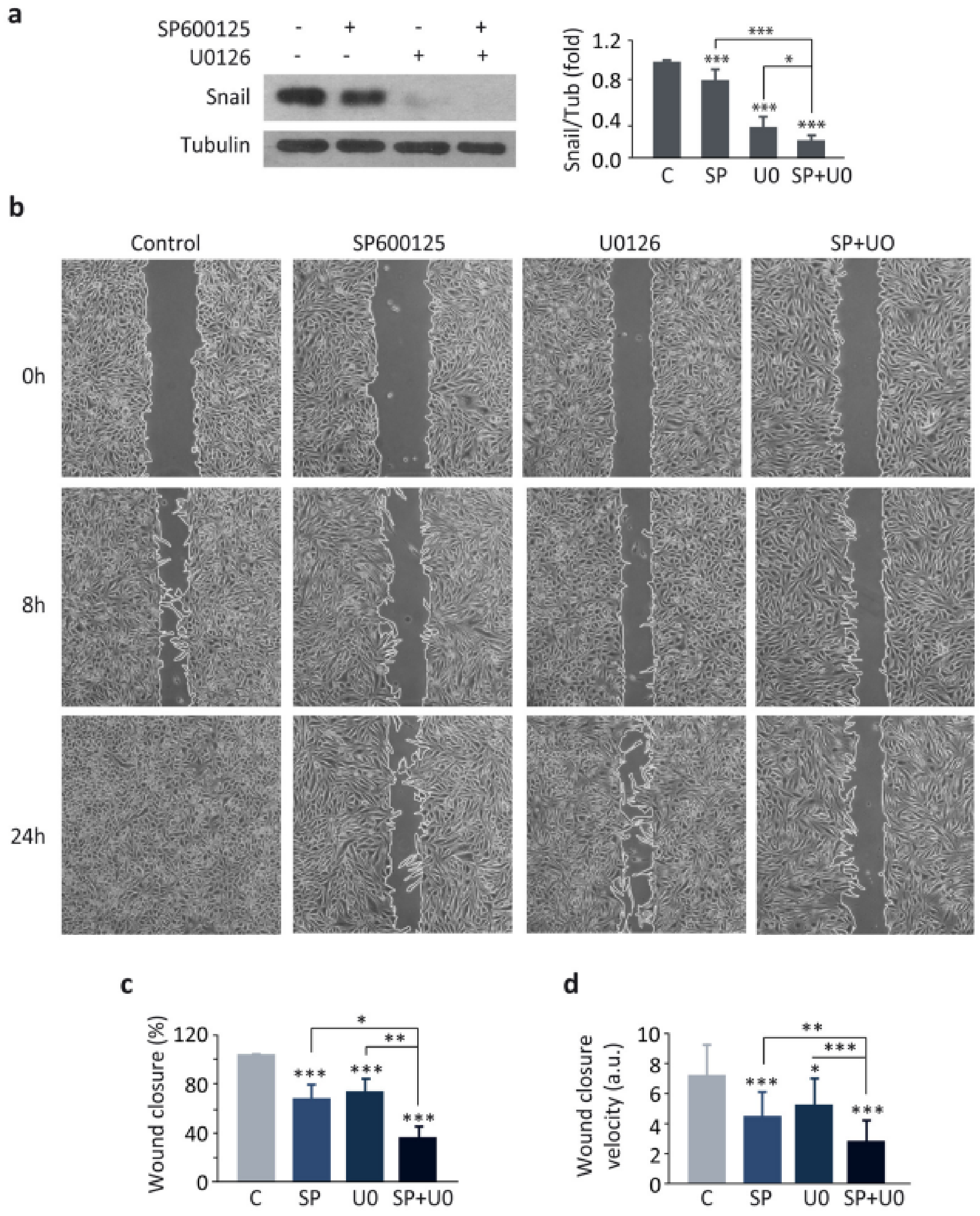

Figure S4. JNK and ERK cooperatively regulate Snail expression and migration in PC3 cells. Cells were incubated in the absence (C) or presence of 10 μM SP600125 (SP, 24 h) and 20 μM U0126 (U0, 48 h). (**a**) Expression levels of Snail and Tubulin were determined by western blotting. (**b–d**) Wound healing assay and measurement of wound closure area and velocity. Data are shown as the mean ± SEM of three independent experiments. For migration assays, pictures are from one representative experiment of three with similar results. Student's t test: * 0.01 < $p < 0.05$; ** 0.001 < $p < 0.01$; *** $p < 0.001$.

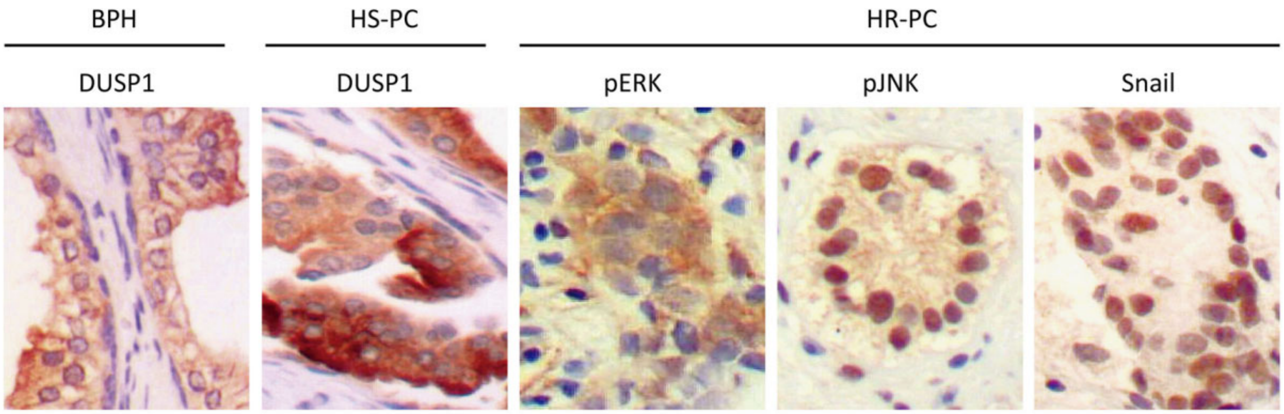

Figure S5. Immunohistochemical analysis showing details of subcellular localization of DUSP1, pERK, pJNK and Snail levels in samples from BPH, HS-PC and HR-PC selected from Figure 7.

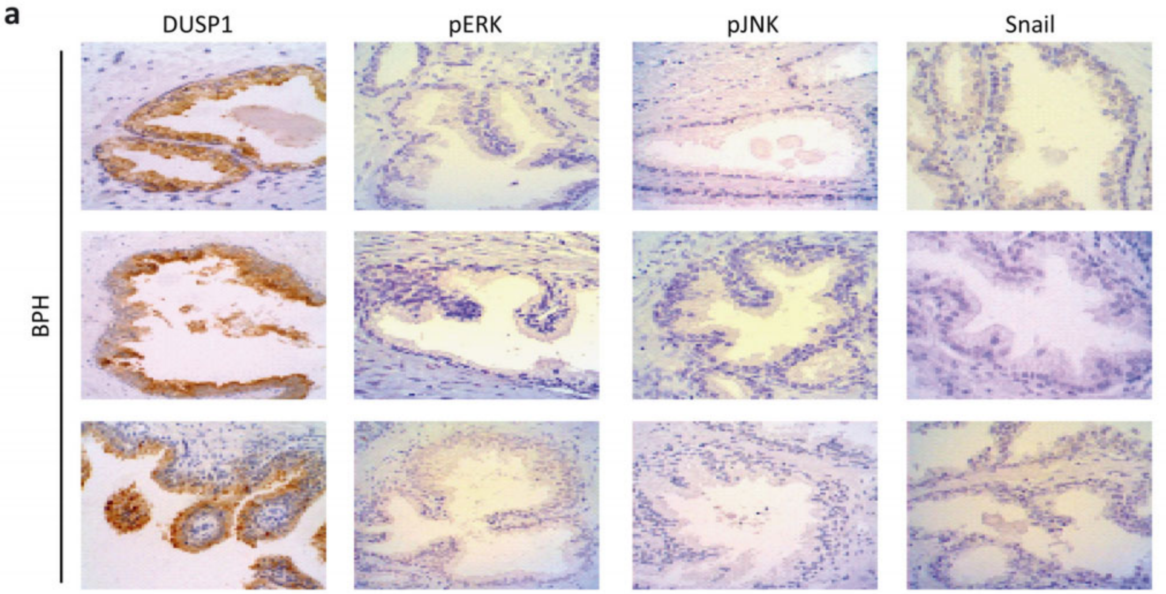

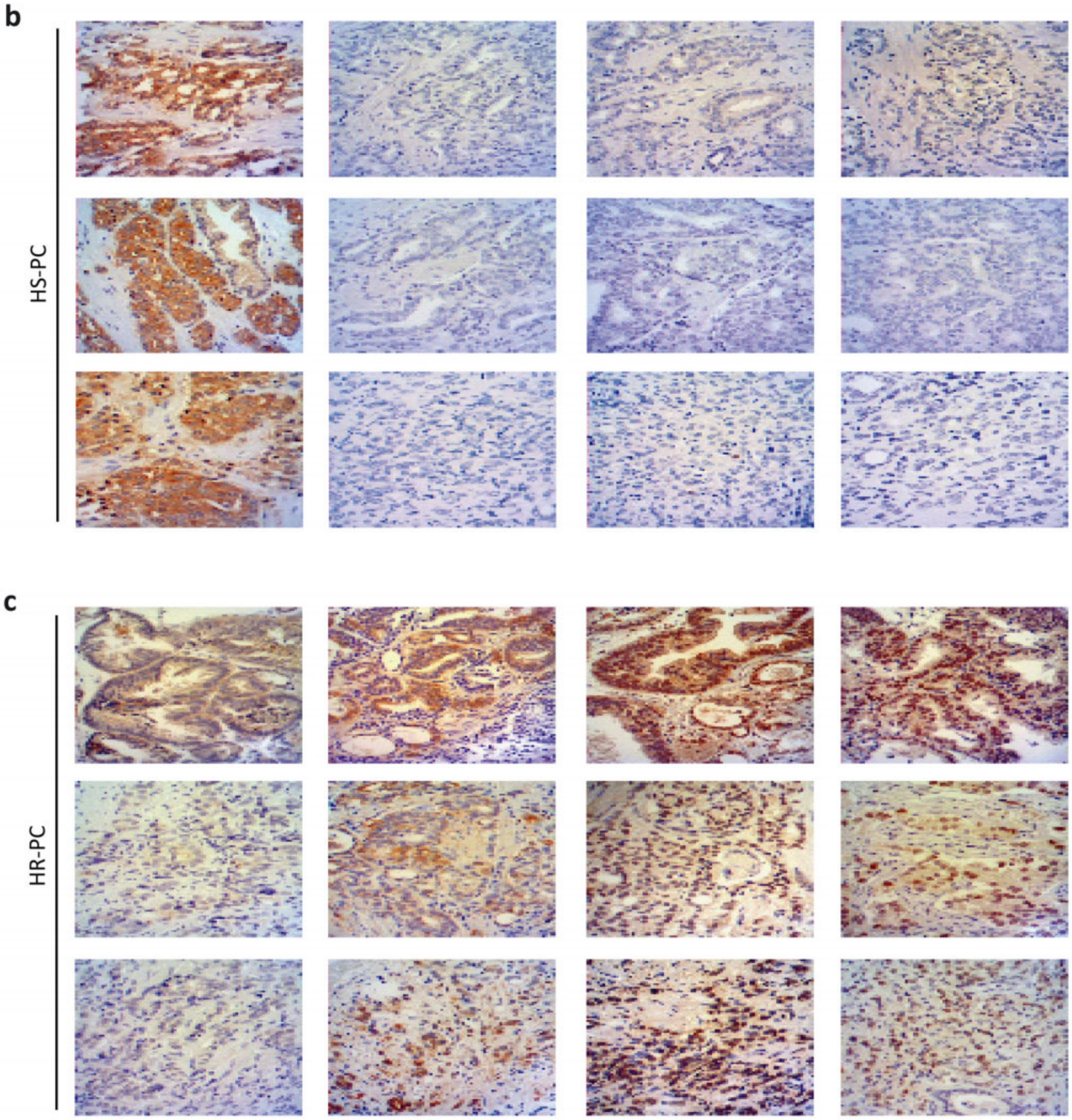

Figure S6. (**a**) Inmmunohistochemical analysis of expression levels of DUSP1, pERK, pJNK and Snail in samples from three different patients diagnosed with BPH. A moderate to strong labelling for DUSP1 was consistently observed, but a negative staining for pERK, pJNK or Snail was detected. In all the images the magnification was 200x. (**b**) Inmmunohistochemical analysis of expression levels of DUSP1, pERK, pJNK and Snail in samples from three different patients diagnosed with HS-PC. A moderate to high labelling for DUSP1 was consistently observed, but a negative staining for pERK, pJNK and Snail was detected. In all the images the magnification was 200x. (**c**) Inmmunohistochemical analysis of expression levels of DUSP1, pERK, pJNK and Snail in samples from three different patients diagnosed with HR-PC. A negative to low labelling for DUSP1 was consistently observed, but a positive staining for pERK, pJNK and Snail was detected. In all the images the magnification was 200x.
